# Spontaneous (?) Abrasion of the Teeth

**Published:** 1882-05

**Authors:** S. H. King

**Affiliations:** Lincoln, Neb.


### ARTICLE VII.

### *Spontaneous (?) Abrasion of the Teeth.*

BY DR. S. H. KING, LINCOLN, NEB.

Every dentist of experience has frequently met with cases of dental disintegration commonly called "spontaneous abrasion." To show, upon etiological principles that no such abrasion exists is the object of this brief paper.

That the character of spontaniety should ever have been given to any disease and found a place as such in our text-books, is as surprising as it is fallacious. One might as well claim a result without a cause, as to imply that a tooth has, or can have in it, normally, the elements of self-destruction. The cases of disintegration alluded to, and which no doubt *you all have* met with in practice, to wit:

the denudation of the cervical portion of teeth of their enamel, and also the crumbling and apparent wearing away of the incising edges of the cuspids and incisors—all have a cause external and entirely foreign to the tooth itself.

First, Let us examine the structure of the enamel. The exterior, or enamel cuticle, is an apparently structureless mass, solid, hard and flinty—upon the perfection of this cuticle almost entirely depends the natural durability of the teeth. Immediately beneath this, lies the enamel proper, composed of hexagonal rods, of solid matter with a slight film or trace of animal matter surrounding each—these rods stand, as it were on end, or run approximately at right angles with the surface of the teeth but in segments, the rods of each section running paralled with each other and the V shaped interstices between the sections filled with similar rods of varying lengths. The chemical composition of the enamel is ninety six and a half per cent. of earthy matter and three and a half per cent. of animal matter. The earthy is principally carbonate and phosphate of lime with traces of fluoride of calcium and phosphate of magnesia.

All these salts are soluble in even dilute acids the enamel cuticle having the strongest resistance to their action. Hence I have no hesitation in saying that in all cases of so-called "spontaneous abrasion" the cause may be traced to acidulous action.

A patient of mine whose teeth I had not seen for two years came to my office a few weeks since, anxious to know what could be done for his teeth. Other dentists had pronounced it a case of "spontaneous abrasion" and beyond cure. Upon examination I found the cervical portion of the incisors and bicuspids denuded of their enamel some of them to the extent of one half of the labial surface. My first remark was—"You eat lemons?" He said he did, and expressed surprise that that was the cause of the trouble.

During my visit in the east a few months since, while making a friendly call upon one of our profession, he nivited my attention to a case then in his office of the abrasion of the incising edges of the six superior single rooted teeth, the disintegration having extended from one-fourth to one-half the distance to the gum, so that upon occlusion an opening of one-eight of an inch was visible between the superior and inferior incisiors.

Upon questioning the patient as to the cause, no satisfactory reason could be found until I inquired what his occupation was? He replied he was employed in a soda or pop factory, and attended principally to charging the soda fountains. The cause was no longer a mystery; the effect of acid fumes was at once settled upon as the solution of the problem and cause of the dissolution of the teeth. A further evidence of the correctness of this theory was that the contour of the abraded edges of the teeth corresponded exactly with the line of the lips protecting the remaining teeth, when the patient parted the lips to breathe through the mouth.

Some authors claim that a slightly acidulous condition is the normal condition of the mouth; this certainly must be an error, for nature would never make such a mistake as to construct useful members like the teeth and at the same time surround them with a merciless destroyer like an acidulous saliva.

I believe the precise normal condition to be neutral. In the many mouths which I have tested, the acidulous were always those in which were found marked modifications of present progressing caries of the dental organs; so that I arrived at the conclusion that a slightly alkaline condition is to be preferred where the golden means of neutrality cannot be maintained. The greatest deficiency in the dental profession seems to be in the direction of etiolgy. In the art of restoration of the lost, and preservation of the decaying, we are far advanced, but how few of us stop to study the cause and intercept it with prevention which is superior to cure.—*Proceedings of the Nebraska Dental Society.*

